# Bradycardia-dependent atrial capture failure after implantation of an atrial leadless pacemaker: Contrasting clinical courses in 2 patients

**DOI:** 10.1016/j.hrcr.2026.05.020

**Published:** 2026-06-10

**Authors:** Yuta Taomoto, Yuichi Ono, Tatsuya Sakamoto, Tetsuya Kishigami, Kenichiro Otomo

**Affiliations:** Department of Cardiology, Ome Medical Center, Ome City, Tokyo, Japan

**Keywords:** Leadless pacemaker, Atrial pacing, Bradycardia-dependent capture failure, Pacing threshold elevation, Device position

**Key Teaching Points**

• Atrial leadless pacemaker implantation may be followed by bradycardia-dependent elevation of the atrial pacing threshold in the early postimplant period.
• When device position remains stable, this phenomenon may be transient and improve spontaneously, allowing conservative observation.
• Persistent threshold elevation may be associated with subtle device positional change; therefore, imaging assessment of device stability is essential when capture failure continues.

Leadless pacemakers have emerged as an alternative to conventional transvenous systems, offering a reduced risk of lead-related complications and pocket infections.^1^^,^^2^ Recently, the introduction of atrial screw-in leadless pacemakers (AVEIR AR; Abbott Medical, Chicago, IL) has further expanded the indications for leadless pacing in patients with sinus node dysfunction.^3^

Bradycardia-dependent elevation of pacing thresholds has been reported in conventional transvenous pacemakers and is known to cause capture failure predominantly at low heart rates.^4^ This phenomenon is believed to be associated with local myocardial injury or inflammation related to lead fixation, and with decreased excitability owing to phase 4 depolarization. Furthermore, rate-dependent changes in pacing thresholds have also been described in ventricular leadless pacemakers (Micra; Medtronic Inc, Minneapolis, MN), suggesting that interactions between the device and myocardium may influence capture stability even in leadless systems.^5^

More recently, transient bradycardia-dependent capture failure has also been reported after implantation of atrial leadless pacemakers, with spontaneous improvement observed during follow-up.^6^ However, the clinical course and underlying mechanisms of this phenomenon remain incompletely understood owing to the limited number of reported cases.

Here, we report 2 patients in whom bradycardia-dependent atrial capture failure developed after implantation of an atrial leadless pacemaker, indicating contrasting clinical courses. In 1 patient, the threshold improved spontaneously, whereas in the other, threshold elevation persisted in association with subtle device displacement. These cases highlight the potential impact of device stability on clinical outcomes.

## Case report

### Case 1

**Figure 1:**
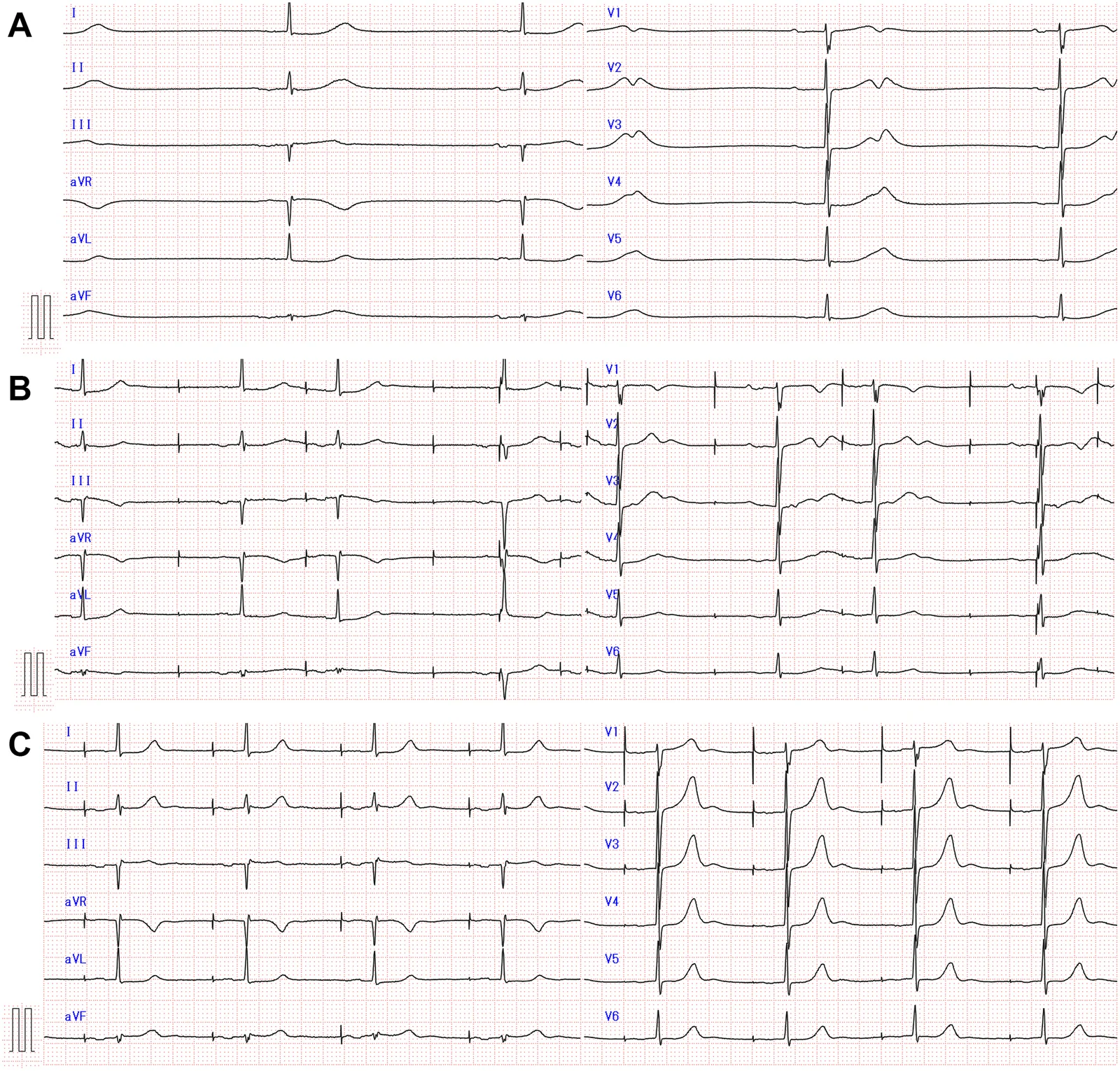
12-lead electrocardiograms in case 1. **A:** At admission. **B:** Postoperative day 1, showing atrial pacing noncapture. **C:** Postoperative day 13, showing stable atrial capture.

A woman aged 86 years presented with a 1-month history of palpitations. Electrocardiography revealed marked bradycardia, and heart rate was approximately 30 beats per minute (bpm) with an ectopic atrial rhythm. The patient was diagnosed with symptomatic sinus node dysfunction (Figure 1A).

Given her advanced age, implantation of both atrial and ventricular leadless pacemakers (AVEIR AR and AVEIR VR) was planned. Transthoracic echocardiography showed left atrial enlargement but no other significant abnormalities.

The AVEIR VR device was successfully implanted without complications. The AVEIR AR device was positioned at the base of the right atrial appendage. Prefixation mapping at a pacing rate of 80 bpm showed pacing thresholds of 2.0V/0.4 ms and 0.75V/1.0 ms. At the end of the procedure, the threshold was 3.25V/0.4 ms; given the expectation of improvement, conservative follow-up was chosen. Initial brady setting was DDD 50–120 bpm.

**Figure 2:**
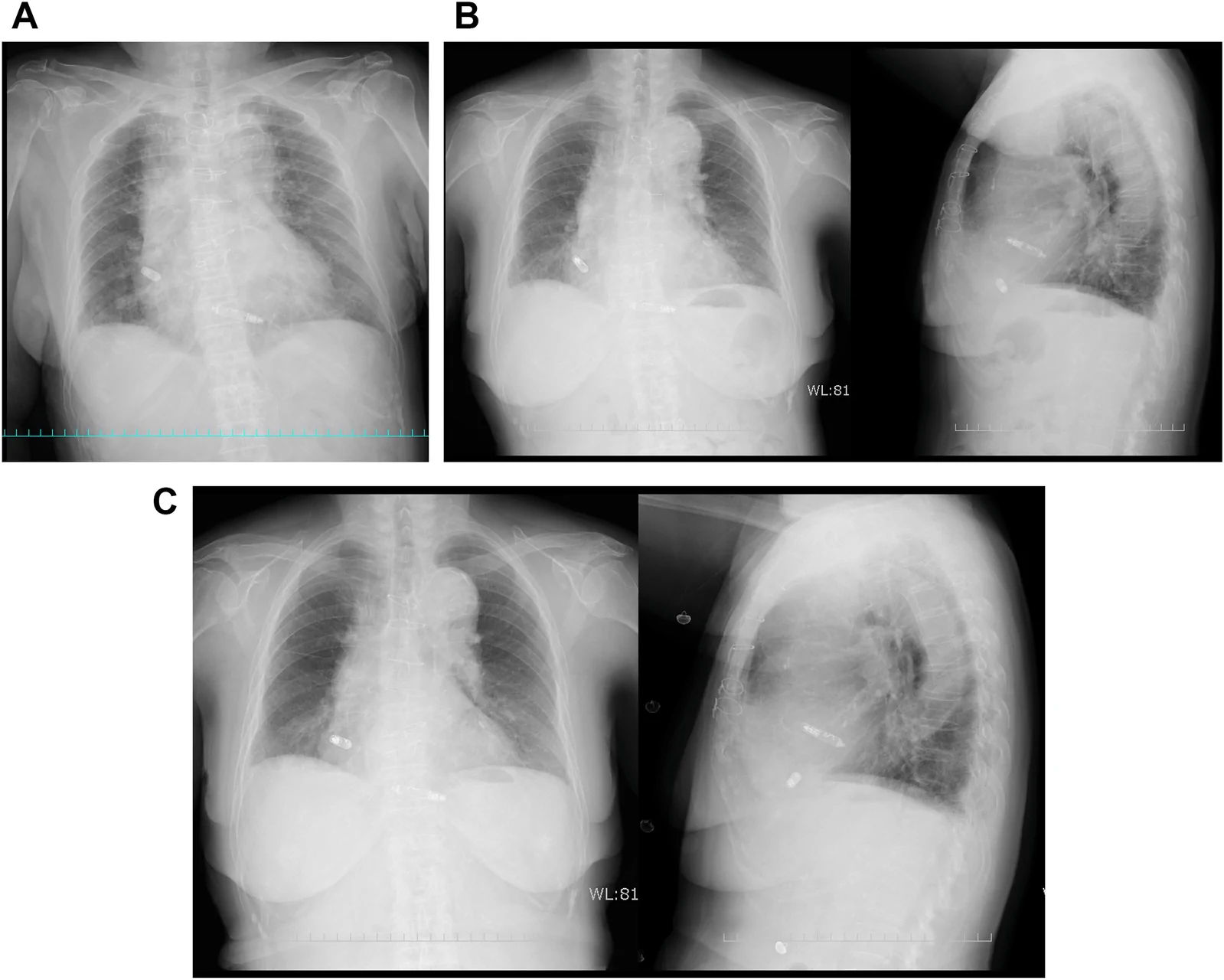
Chest radiographs in case 1. **A:** Implantation day. **B:** Postoperative day 1. Left panels show anteroposterior views, and right panels show lateral views. **C:** Postoperative day 13. Device position remained unchanged. Left panels show anteroposterior views, and right panels show lateral views.

On postoperative day 1, atrial pacing noncapture was observed on electrocardiography (Figure 1B). Device interrogation revealed rate-dependent threshold variation: 2.75V at 60 bpm, 2.25V at 70 bpm, 2.0V at 80 bpm, 1.5V at 90 bpm, and 1.5V at 100 bpm, indicating higher thresholds at lower pacing rates. Chest radiography on the day of implantation and the following day showed no apparent change in device position (Figure 2A and 2B).

The pacing threshold gradually improved over time. By postoperative day 4, a downward trend was observed, and by day 13, thresholds had decreased to 1.25V at 50 bpm and 1.0V at 60–90 bpm, with continued improvement thereafter. Impedance remained stable throughout follow-up (Table 1, case 1). Chest radiography on day 13 confirmed stable device position (Figure 2C). Ultimately, stable atrial capture was achieved (Figure 1C), and neither device retrieval nor reimplantation was required. These findings suggest that the threshold elevation in this case was transient and likely related predominantly to early postimplant myocardial response, although subtle mechanical factors could not be completely excluded.

**Table 1:** Serial atrial pacing thresholds and impedance during follow-up

Case 1						
Pacing Rate	Premap	Implant d	POD 1	POD 4	POD 13	POD 47
50					1.25V/0.4 ms	
60			2.75V/0.4 ms	3.25V/0.4 ms	1.0V/0.4 ms	0.5V/0.3 ms
70			2.25V/0.4 ms	2.25V/0.4 ms	1.0V/0.4 ms	0.5V/0.3 ms
80	2.0V/0.4 ms∗ 0.75V/1.0 ms	3.25V/0.4 ms	2.0V/0.4 ms	1.5V/0.4 ms	1.0V/0.4 ms	0.5V/0.3 ms
90			1.5V/0.4 ms	1.25V/0.4 ms	1.0V/0.4 ms	0.5V/0.3 ms
100			1.5V/0.4ms			
Output		4.5V/0.4 ms	4.5V/0.4 ms	3.5V/0.4 ms	2.5V/0.4 ms	2.5V/0.4 ms
Impedance		270Ω		320Ω	330Ω	310Ω

Case 2						
Pacing Rate	Premap	Implant d	POD 1	POD 4		
50						
60			6.0V/0.4 ms			
70			1.0V/0.4 ms			
80	1.0V/0.4 ms	0.5V/0.4 ms				
90			0.75V/0.4 ms			
100				Noncapture (6.0V/1.0 ms)		
Output		2.5V/0.4 ms	3.5V/1.0 ms			
Impedance	270Ω	290Ω	370Ω	340Ω		

Case 1: Bradycardia-dependent threshold elevation improved over time.

Case 2: Persistent threshold elevation despite maximal output.

Output data on POD 4 were omitted because the device had been switched to ventricular pacing mode at that time.

POD = postoperative day.

∗ Thresholds were measured using pulse widths of 0.4 and 1.0 ms.

### Case 2

**Figure 3:**
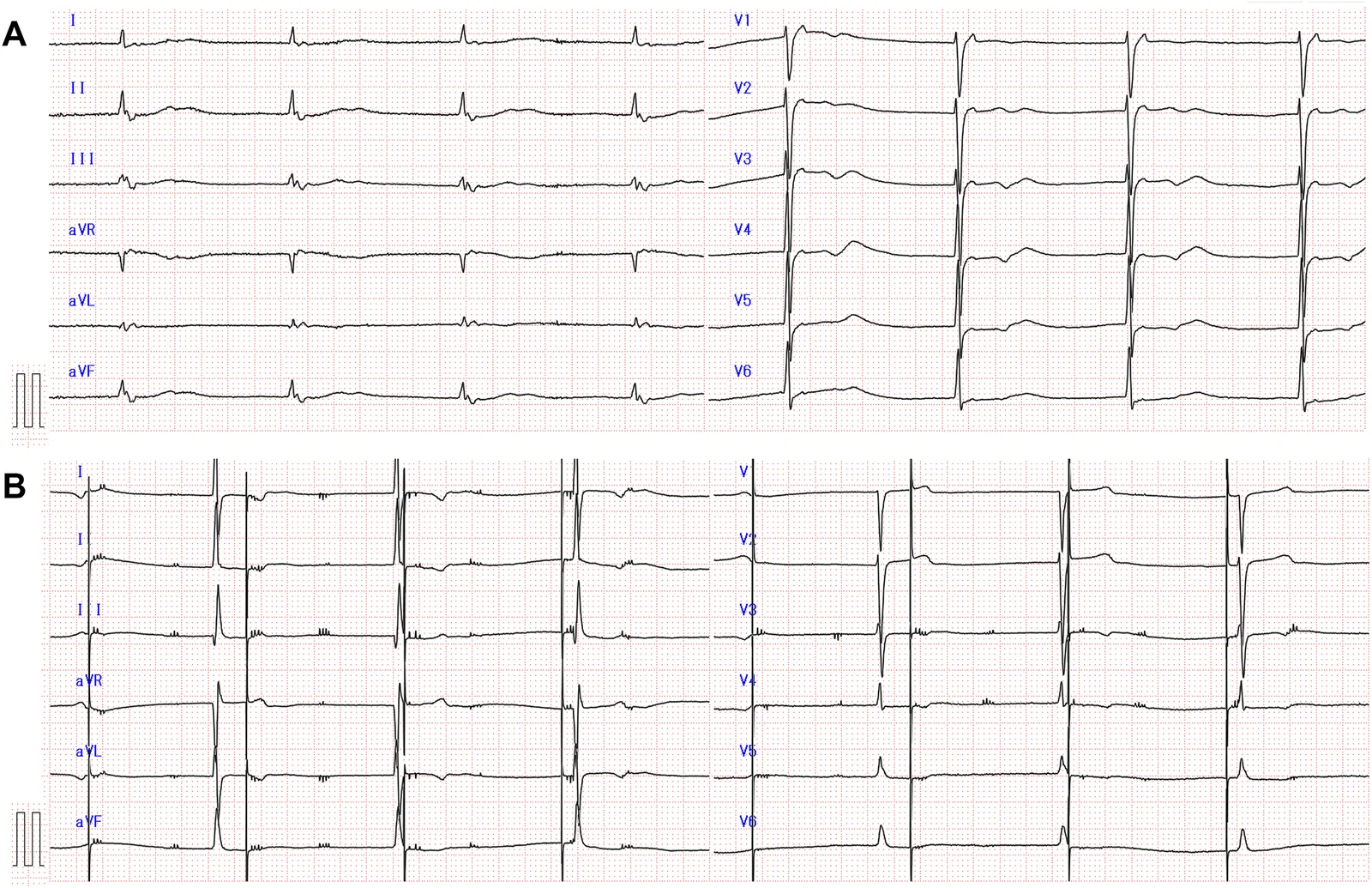
12-lead electrocardiograms in case 2. **A:** Junctional rhythm at 48 beats per minute (bpm) on admission, consistent with sinus node dysfunction. **B:** On postoperative day 4, atrial pacing noncapture was observed at a pacing rate of 50 bpm despite high-output pacing at 6.0V/1.0 ms. After transient atrial tachycardia, atrial pacing noncapture persisted despite high-output pacing of 6.0V at 1.0 ms at a pacing rate of 100 bpm.

A woman aged 84 years presented with persistent exertional palpitations. Electrocardiography indicated a junctional rhythm at 48 bpm, and the patient was diagnosed with symptomatic sinus node dysfunction (Figure 3A).

After treatment for concomitant heart failure with temporary pacing support, implantation of AVEIR AR and AVEIR VR devices was performed. Transthoracic echocardiography revealed left atrial enlargement but preserved left ventricular function and no significant valvular disease.

The AVEIR VR device was successfully implanted. The AVEIR AR device was positioned at the base of the right atrial appendage. Prefixation mapping at a pacing rate of 80 bpm revealed a threshold of 1.0V/0.4 ms, which improved to 0.5V/0.4 ms after screw fixation.

Despite favorable initial parameters, threshold elevation was observed on postoperative day 1. The threshold was 6.0V/0.4 ms at 60 bpm, 1.0V/0.4 ms at 70 bpm, and 0.75V/0.4 ms at 90 bpm, again showing rate dependence. On postoperative day 4, atrial pacing noncapture was also observed at a pacing rate of 50 bpm despite high-output pacing at 6.0V/1.0 ms (Figure 3B). Transient atrial tachycardia developed in the patient, and atrial pacing noncapture persisted even at 6.0V/1.0 ms at a pacing rate of 100 bpm. Impedance remained stable (Table 1, case 2).

**Figure 4:**
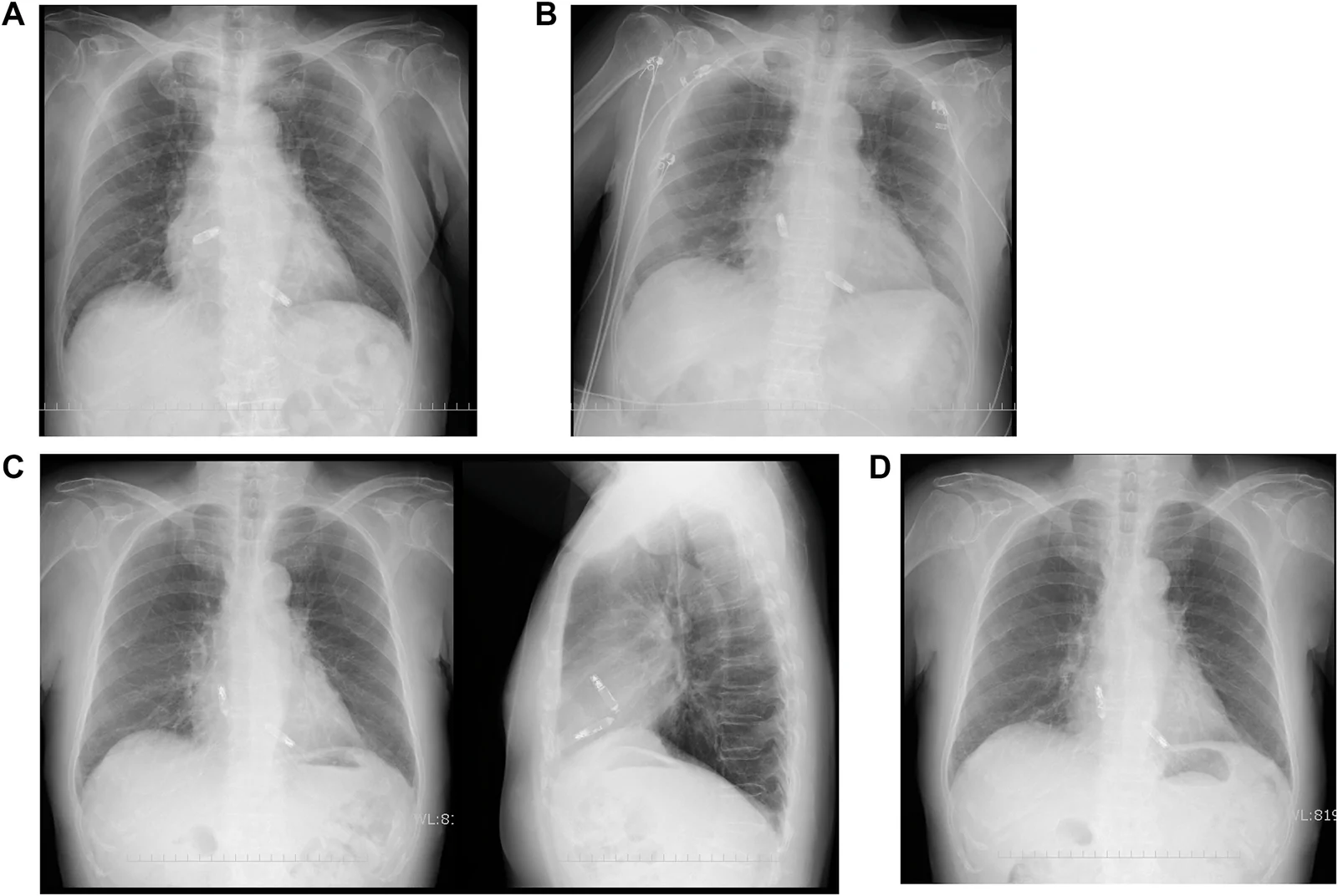
Chest radiographs in case 2. **A:** Implantation day. **B:** Postoperative day 1. **C:** Postoperative day 2. Left panels show anteroposterior views, and right panels show lateral views. **D:** Postoperative day 4. Subtle device displacement was observed.

Chest radiography revealed subtle displacement of the device compared with its initial position (Figure 4). Considering the patient’s advanced age and clinical condition, device retrieval or reimplantation was not performed, and the patient was managed with ventricular pacing mode. Atrial tachycardia persisted, precluding reassessment of threshold recovery. The persistent threshold elevation was considered likely related to minor device displacement affecting electrode–myocardial contact.

## Discussion

This report shows that bradycardia-dependent atrial pacing threshold elevation can occur after implantation of an atrial leadless pacemaker and that its clinical course may vary depending on device stability. Although similar cases have been previously reported, our cases uniquely indicate contrasting clinical courses, suggesting that device stability may influence whether threshold elevation is transient or persistent.

**Figure 5:**
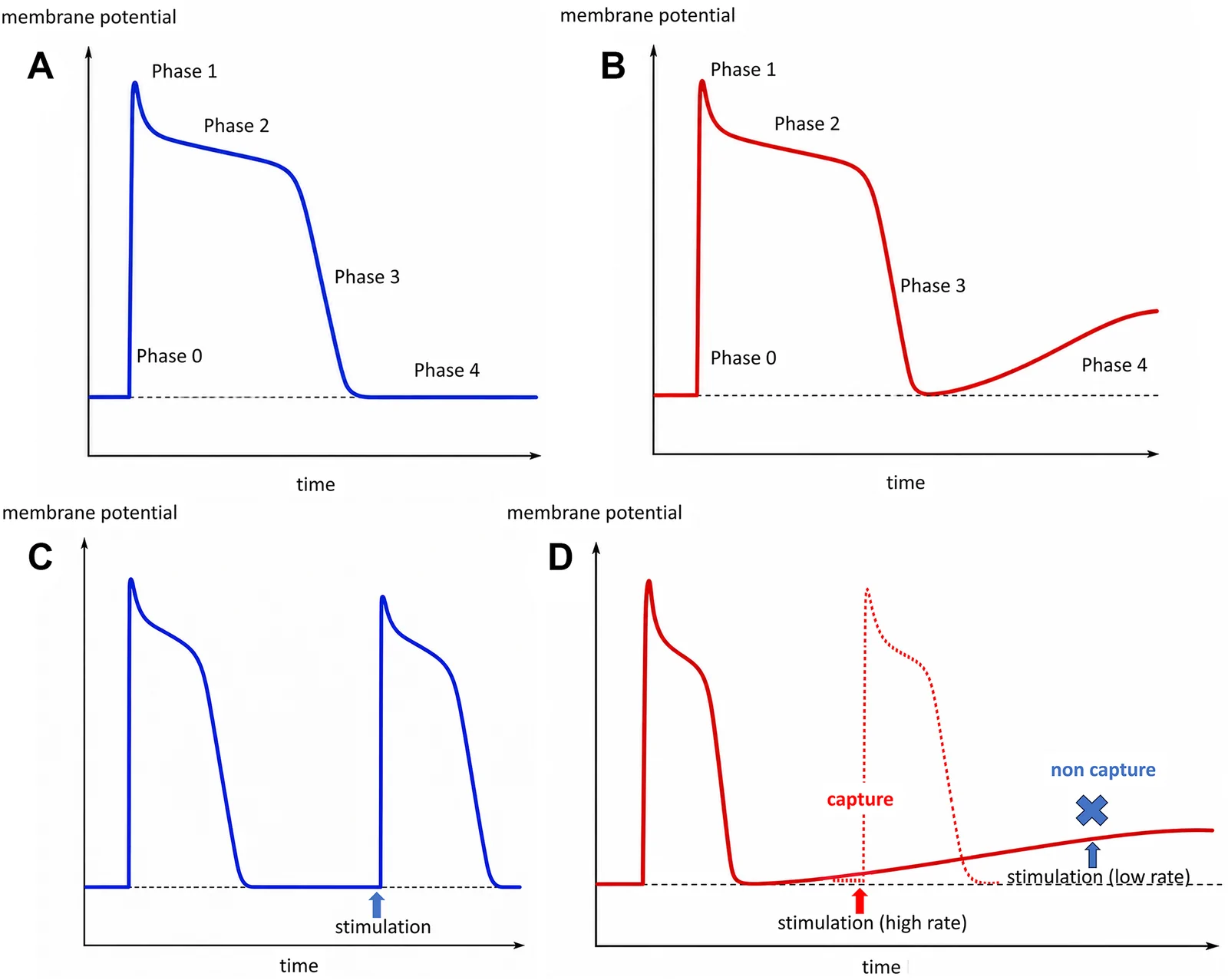
Schematic illustration of phase 4–related loss of capture as a potential mechanism of bradycardia-dependent noncapture. **A:** Normal myocardial action potential with a stable resting membrane potential during phase 4. **B:** Schematic representation of phase 4 depolarization, in which the membrane potential gradually becomes less negative during prolonged diastolic intervals. **C:** Under normal conditions, pacing stimuli delivered after sufficient repolarization yield successful capture. **D:** Proposed mechanism of bradycardia-dependent noncapture. During longer cycle lengths, progressive phase 4 depolarization leads to partial depolarization of the local myocardium, reducing sodium channel availability and myocardial excitability. As a result, low-rate pacing stimuli may fail to generate a propagated action potential, whereas higher-rate pacing delivered before substantial phase 4 depolarization can still achieve capture.

Previous reports have described transient bradycardia-dependent threshold elevation after atrial leadless pacemaker implantation, with spontaneous improvement occurring within several days.^6^ This phenomenon has been attributed to temporary myocardial injury or inflammation associated with device fixation, leading to transient changes in excitability. In addition, phase 4–related loss of capture in the setting of atrial myocardial injury or inflammation may also contribute to this phenomenon^7^ (Figure 5).

In case 1, device position remained stable; impedance was unchanged, and pacing thresholds improved over time. These findings are consistent with a reversible biological mechanism, such as local myocardial inflammation or injury. However, we cannot completely exclude the possibility that subtle changes in the device–myocardial interface contributed to gradual threshold improvement over time.

In contrast, case 2 showed persistent threshold elevation accompanied by subtle device displacement. Although leadless systems eliminate traditional leads, even minimal changes in device position or contact angle may significantly affect the electrode–myocardial interface and thus capture characteristics. Factors such as atrial contraction, body movement, or arrhythmias may contribute to such microdisplacement.

These observations suggest that bradycardia-dependent threshold elevation after atrial leadless pacemaker implantation is not a uniform phenomenon. When device position is stable, the condition may be transient and self-limiting. However, when device displacement occurs, threshold elevation may persist and lead to ongoing pacing failure.

From a clinical perspective, early pacing failure after implantation does not necessarily mandate immediate reintervention if device position is stable and impedance remains within normal range. Careful observation may be appropriate in such cases. Conversely, persistent threshold elevation should prompt imaging evaluation to assess device position, given even subtle displacement may influence management decisions.

Further accumulation of cases is needed to clarify the mechanisms and optimal management strategies for this phenomenon. Nevertheless, our findings underscore the importance of considering both biological and mechanical factors when evaluating pacing instability in atrial leadless pacemakers.

## Conclusion

Bradycardia-dependent atrial pacing threshold elevation can occur after implantation of an atrial leadless pacemaker, and its clinical course may depend on device stability. When device position is stable, threshold elevation may be transient and improve over time. In contrast, persistent elevation may be associated with device displacement. Assessment of device position is essential when early pacing failure is observed, and this factor should guide management decisions. Recognition of this phenomenon may help avoid unnecessary early reintervention in selected patients with stable device position.

## Funding Sources

This research did not receive any specific grant from funding agencies in the public, commercial, or not-for-profit sectors.

## Disclosures

The authors have no conflicts of interest to disclose.
